# A Much-Neglected Branch of Dermatology

**Published:** 1913-05

**Authors:** W. W. Quinton

**Affiliations:** Buffalo


### A Much-Neglected Branch of Dermatology.

BY W. W. QUINTON, M.D.
Buffalo

IT is certainly true that the medical profession does not give to cosmetic treatment the attention which it deserves. The result is that many persons are driven to the so-called "dermatologists" who advertise freely in our daily press, and who reap thereby a plentiful harvest.

For example, one individual advertises as follows: Superfluous Hair and Facial Blemishes Removed by Multiple Electrolysis. Wasted, Hollow Cheeks and other Imperfections of Features Corrected by the Famous Electro Vacco Process."

The newspapers are full of paragraphs exploiting nostrums for the cure of wrinkles, superfluous hair, and for the care of the scalp, and for every conceivable eruption.

It seems to me that the medical profession is deeply responsible for this state of affairs, because, by our indifference, we drive many of our patients to the quack and the charlatan. Beauty is a priceless possession, and when we are called upon to prescribe for women who are suffering from some disfiguring condition, we must either accomplish the result they desire or else expect that the case will pass on to some irregular practitioner.

The average physician in general practice regards these cases too lightly. He seems to be impelled by the fear that he may earn the despised title of "Beauty Doctor" if he makes any determined and serious attempt to handle such cases. The result is that most women seek out, by preference, the man who sports the initials "E. D." after his name, knowing that they will be taken seriously and treated accordingly, and fondly believing that the treatment will be scientific. If the prospect of relief seems hopeful to her, a woman is not apt to forego a course of treatment simply because a practitioner has no college degree and is not entitled to print "M. D." on his professional cards. When they suffer with eczema, psoriasis, lupus, etc., they are likely to consult one of the regular profession without delay, but if it be a case of superficial blemish, "bad complexion," "pimples," or some disease of the scalp, nine times out of ten it is the quack who gets the case. How many women slip secretly into such establishments in search of treatment which they should receive openly, at the hands of a regular practitioner? How many such women gain the relief which they seek? How many of them are marred for life as a result of ill-judged, bungling treatment?

The futility and in many cases tragedy of trusting to quack treatment is well known. One of many cases which have come to my notice is a good illustration. A woman with a poor complexion—for which malnutrition and ill health were directly responsible—visited a "beauty specialist" who advertises freely and has an extensive clientele. For the sum of two hundred dollars this specialist guaranteed to transform her skin into one of youth and beauty. The procedure consisted in applying an acid or a strong solution of bichloride, with an after dressing of strips of zinc oxide plaster. This induced a rapid scaling and at the end of ten days when the plaster was removed it revealed a new skin as delicately pink and white as that of a newborn babe. Unfortunately, at the end of a week another lightning-like transformation occurred and soon after the woman's complexion became worse than it ever had been before—so bad in fact that eventually she had recourse to a regular dermatologist for treatment and cure. Nothing remained of the original quack treatment but the memory of intense and prolonged pain and the stub in the check book with its two hundred dollar notation.

It will be well to make note, first of all, in the treatment of this subject, that applied cosmetics and the principles of hygiene go hand in hand. "Wasted, Hollow Cheeks and other Imperfections of the Features" will never be corrected by local means alone—even the "Famous Electro Vacco Process' will fail in such instances.

Here, then, is where the non-scientific man commits a gross error. We cannot expect the face to remain in a normal condition of health and beauty when every other part of the body is in an abnormal state. Our first step must be to search out and remove the causes which are reacting to the detriment of the patient's system. This involves a thorough physical examination. If we find, for example, that an auto-intoxication is responsible for a skin eruption, we must treat the patient in such a way that the poisoning will cease. Sometimes we find one local condition responsible for another, as, for instance, an acne rosacea which is kept up by hypertrophic or atrophic conditions in the nose, which disturb the circulation. Or we may find that an unhealthy scalp is responsible, to a marked extent, for a disfiguring eruption on the face.

We regulate the diet in all cases—carefully prescribing such articles of food as each individual case requires and impressing the patient with the great importance of this measure. Tonics are essential in many cases, but what we must emphasize in our treatment is the careful observance of hygienic rules. The proper kind and amount of food, regular hours, rest at night, exercise and fresh air, plenty of pure water to drink, regulation of the bowels and proper bathing. For the foundation of all beauty of face and form is health, and this must constitute the basis for our treatment. To it we add, as the case may require, various local measures.

Facial massage has its place in cosmetic treatment when applied intelligently. A massage powder may be used if desired.

We may use facial applications, either in the form of lotions or ointments, which vary in strength from the very mildest, soothing preparations, to those which have a profound exfoliative action on the skin. But we must never neglect the foundation of health which is so necessary if we wish the improvement to be permanent.

Not the least important in the treatment of acne is the local treatment of each pustule and the removal of every comedone. This will be facilitated by a preliminary steaming of the face by means of a dermothermostat—or at least a preliminary bathing of the face with soap and hot water. We soften the water used for washing purposes by adding borax or camphor and benzoin.

There are a variety of neutral soaps on the market which are excellent in these cases. Hebra's alkaline soap spirit may often be used to advantage. Too frequent washing of the skin, particularly in the cases of those whose skin is over-sensitive, will do harm. The importance of thoroughly drying the skin after washing cannot be overestimated. Sponging with alcohol immediately after bathing will benefit some cases.

The removal of small blemishes—warts, moles, new vessel formations, etc.,—may readily be affected by electrolysis, which, in the hands of the skilful operator, will give excellent cosmetic effects.

In the treatment of hypertrichosis—superfluous hair—electrolysis offers a safe and sure treatment. If the technique be good, no scarring will result and the skin will be left in excellent condition. The use of the X-ray, with the Cornell tube, also offers a safe treatment for this condition and one which is preferred, by many operators, to the use of the electric needle.

The care of the hair is of great importance. The scalp suffers either from a lack of attention on the one hand or from too much of the wrong kind of treatment on the other. Most of us are convinced that the general health has a direct bearing on the condition of the scalp, so our first effort must be to correct any systemic disorders which may exist, and to bring the patient's bodily functions as near normal as possible. When we turn to local treatment most of us are non-plussed by the multitudinous prescriptions which our text books reveal. We must not allow this to confuse us for it will be found that each case must be studied and treated individually, for no one treatment will apply equally well to every case.

The condition must be treated early, for when the hair has been lost and has been replaced by a lanugo growth, and when marked changes have occurred in the sebaceous glands, the prognosis is most unfavorable. Women are quick to note a seborrhoea of the scalp and generally seek advice and treatment without delay. Too often they turn to the quack, or to quack nostrums which are so freely advertised in our daily press. This is the surest way to lose time, money and hair, for if ever an effection required careful, scientific attention, this one undoubtedly does. Reverse the usual "before" and "after" pictures which accompany so many advertisements and you will have a faithful representation of the result of quack remedies and treatment.

In a majority of cases—which are not too far advanced—we may look for favorable results if our patients will faithfully carry out treatment. The scalp must be shampooed at regular intervals and in the right way; scientific massage is valuable, and regular brushing and combing of the hair should be insisted upon. We must prescribe lotions or ointments with exact reference to the condition of the scalp and hair.

The effect of the mind on cutaneous disturbances, must not be lost sight of. Even so small a matter as the loss of a night's rest will leave its mark on the skin; a much more serious effect will be produced if the patient allows her mind to dwell upon her symptoms. Here psychotherapy has its place, quite as much as in the graver bodily disturbances. Often a nervous breakdown has its incipiency in just such a trifling cause as a "bad complexion." The incidental worry, loss of sleep, disinclination to appear in public, etc., soon establish the vicious circle. The general health suffers, the "bad complexion" becomes worse, and the worry and its train of attendant symptoms are exaggerated. It only requires a jovial physician to make light of the matter and prescribe a harmless "placebo," and another convert and faithful worshipper is presented to the advertising quack.

It is impossible to more than touch upon a few of the points of cosmetic treatment in a single article, and it was not my intention to attempt more. If this article has the effect of arousing some interest in a much neglected and almost overlooked branch of dermatology, then it will have served its purpose. The point is that we, as scientific men, are in a much better position to administer cosmetic treatment than the hairdresser, the barber, or the charlatan. What little knowledge they possess, is ours also, and in addition we have the greater advantage of collegiate and hospital training.

To improve the medical situation in this country we must educate the public. I venture to say that if every physician would take up arms and join the American Medical Association in its fight against medical fakes and nostrums, and make an earnest attempt in his immediate locality to put the public in possession of the facts, then, indeed, it would not be long before the patent medicine men would be forced to adopt some more honest if less lucrative form of employment.

The sooner we realize that cosmetic treatment is a part and parcel of scientific dermatology, the better for the ultimate standing of that special branch of medicine. Certainly I know of no other class of cases where scientific management and painstaking technique are more urgently required, both of which are too much to ask of the barber and the quack-electrician.

If every physician would do his duty in the interests of cosmetics, that branch of dermatology would soon assume its proper place in medical circles, to the great dismay of the quacks and to the greater advantage of a long-suffering public.

W. W. QUINTON, M. D.

---

POLYMYOSITIS WITH MULTIPLE LIME DEPOSITS. Sara Welt Kakels, *Paediatrics,* March, 1913. The case was in a boy of five, with no significant family history, except that he had had ophthalmia neonatorum with recovery. Lameness, painful swellings, an eight-day exanthema diagnosed as scarlet fever, two years ago, subsequently, occasional urticarial attacks. Lime demonstrated in subcutaneous tissues and probably present in muscles.

---

HYDROCHLORIC ACID FORMATION IN THE STOMACH. J. Lopez-Suarez, *Biochem. Zeit.,* 1912, No. 46 (cf. *Jour. of the A. M. A.,* January 25, 1913, page 284 and March 15, page 838) claims that the parietal cells of the gastric tubules are poor in chlorids and are neutral or even alkaline even when HCl is being secreted. The chief cells contain more chlorin than the parietal, which have hitherto been considered oxyntic cells. The fundus is known to produce HCl more than other parts of the stomach and actually contains a slight relative excess of chlorin.

